# Hemicrania Continua Headache in a Veteran with Posttraumatic Stress Disorder and Major Depressive Disorder without Traumatic Brain Injury

**DOI:** 10.1155/2012/937217

**Published:** 2012-06-03

**Authors:** Brandon A. Kohrt, Erica Duncan

**Affiliations:** ^1^Department of Psychiatry and Behavioral Sciences, The George Washington University, Washington, DC 20037, USA; ^2^Atlanta Veterans Administration Medical Center, Decatur, GA 30033, USA; ^3^Department of Psychiatry and Behavioral Sciences, Emory University School of Medicine, Atlanta, GA 30322, USA

## Abstract

Hemicrania continua is a headache characterized by chronic unremitting unilateral pain associated with ipsilateral autonomic findings. This type of headache responds to high-flow oxygen and indomethacin. This case report describes a male veteran with posttraumatic stress disorder (PTSD) and major depressive disorder who suffers from comorbid hemicrania continua. The psychiatric symptoms were recalcitrant to psychopharmacological intervention. However, when the patient's hemicrania continua was treated appropriately, the patient's psychiatric symptoms also abated. This case demonstrates the need to address physical comorbidities that may exacerbate psychiatric disorders, such as PTSD.

## 1. Introduction

Headaches are a common somatic complaint among persons with psychiatric conditions [1], and persons with headaches when screened have a greater prevalence of psychiatric symptoms compared with the general population [2]. There is an extensive literature on headaches among persons with depression and anxiety [1]. Among persons with posttraumatic stress disorder (PTSD), most descriptions of patients with headaches occur in the context of comorbid traumatic brain injury (TBI) [3]. However, there is a dearth of literature discussing headaches among PTSD patients without TBI. The proper identification and appropriate treatment of headache types among patients with PTSD are important as headache palliation may influence resolution of PTSD symptoms. The case below describes the presence of a rare form of new-onset persistent daily headache, hemicrania continua [4], in a veteran with PTSD.

## 2. Case Report

A 50-year-old African-American veteran with a history of PTSD and severe recurrent major depressive disorder (MDD) presented to the psychiatric emergency room with complaints of suicidal ideation and exacerbation of traumatic reexperiencing in the form of flashbacks of dismembered body parts witnessed during his experience in the first Gulf War. The patient was diagnosed with PTSD and MDD nine years after his traumatic experience. He had suffered from flashbacks, nightmares, avoidance of memories, avoidance of military personnel who reminded him of the event, insomnia, and hyperstartle. In addition, he had prolonged periods of depressed mood, anhedonia, feelings of worthlessness, and psychomotor retardation. These symptoms of depression had predated his military experience. He also had periods of suicidal ideation since his military service; however, he had never attempted suicide. The patient also reported waxing-waning headaches that began three years prior to this emergency room presentation. He reported that his flashbacks, nightmares, and suicidal thoughts worsened during these headaches. The patient's traumatic exposure did not include a head injury. The patient had not been directly exposed to any concussive blasts, had no history of loss of consciousness, and denied any other history of head injuries. He did not meet criteria for traumatic brain injury.

The patient was admitted for inpatient psychiatric treatment, which included continuing his outpatient medications of citalopram 40 mg for treatment of MDD and PTSD and prazosin 5 mg for PTSD-associated nightmares. The patient had been taking acetaminophen and gabapentin for headaches. These were discontinued upon admission because the patient reported no pain relief from these medications. The patient was changed to ibuprofen 800 mg as needed for headaches. During the first week of hospitalization, the patient spent most of his days fully submerged under bedcovers in his room. He wanted limited interaction with other veterans as they reminded him of his traumatic experience. He complained of daily headaches and reported exacerbation of the headache with light and noise; the ibuprofen contributed minimally to palliation.

The ibuprofen was tapered and then discontinued because of concern for nonsteroidal anti-inflammatory drug (NSAID) rebound. He was started on sumatriptan because of the medical team's presumption that the patient may be having migraines. The patient showed no relief with sumitriptan. Further discussion with the patient revealed that his headaches had been constant for greater than eight months, were localized to the left side, and associated with left-sided rhinorrhea and lacrimation. At the beginning of the third week of hospitalization, the patient's headache was discussed with a neurology consultant who recommended trying high-flow oxygen or indomethacin. The neurology consultant suggested inclusion of hemicrania continua in the differential diagnosis. The patient was given a 15-minute trial of oxygen at eight liters per minute.

The patient reported that the headache abated significantly within two minutes of oxygen treatment and did not recur. By the next morning he reported significant improvement in his depression and PTSD symptoms, which had been recalcitrant over the prior three weeks. The patient was discharged after three weeks of hospitalization with no headache and significant improvement in his depression and reexperiencing symptoms.

The patient returned to the psychiatric emergency room one week later reporting that the headache had recurred, and he was having traumatic reexperiencing of “bodies, blood, and horror. For three days, I was doing good, but then I just became depressed again and had the nightmares of dead bodies.” The patient was then started on indomethacin 25 mg three times daily. After one day of indomethacin, the patient reported abatement of the headache. He remained pain-free for an additional week on indomethacin. Moreover, the patient reported that his depression and PTSD symptoms were well controlled when the headaches were treated.

Unfortunately, the patient did not adhere to the indomethacin regimen after discharge. He reappeared in the psychiatric emergency department two months after discharge reporting that the headaches had recurred and increased in intensity over the past three weeks. He said that his nightmares, depressed mood, and suicidal thoughts crescendoed with the increasing headache intensity. He was treated again with high-flow oxygen and indomethacin, which abated the headache. He was discharged after a three-day inpatient hospitalization. The course of the patient's headaches and psychiatric symptoms after that hospitalization are not known.

## 3. Conclusion

This case study illustrates the importance of a thorough headache screening among patients with PTSD including in the absence of TBI. Migraine, cluster, and tension headaches may commonly occur with PTSD. Rarer forms of headaches such as hemicrania continua should also be considered in patients with chronic unremitting unilateral headaches associated with ipsilateral autonomic findings, which are the hallmark symptoms of hemicrania continua [4]. The pathophysiology of hemicrania continua, as observed through positron emission tomography, is characterized by involvement of the posterior hypothalamus contralateral to the pain as well as the dorsal pons and ventral midbrain ipsilateral to the pain [5].

For some trauma survivors, physical pain may act as a stimulus for traumatic reexperiencing. Therefore, control of physical complaints, such as headaches, may lead to abatement of PTSD symptoms. This also is true among trauma survivors in non-Western cultural groups for whom headaches and other somatic complaints may be common comorbidities with psychiatric disorders [6–8]. The HADStress screen has been developed as a PTSD screen that uses four somatic complaints: headaches, appetite changes, dizziness, and sleep problems, and this instrument has good psychometric properties and cost effectiveness in trials of screening Ethiopian and Somali refugees in the United States [9, 10].

This case illustrates that effective treatment of headaches may contribute to a reduction of posttraumatic and other psychiatric symptoms. However, without ongoing control of somatic complaints, there is a risk of relapse of psychiatric symptoms, such as the depression and PTSD symptoms in this patient. This patient may have benefited from outpatient psychotherapy to promote adherence with a treatment that effectively controlled both his psychiatric and somatic complaints. In general, outpatient and community-based management of somatic complaints is needed in addition to acute control of somatic complaints in an inpatient setting. Therefore, outpatient psychiatric health workers should screen for somatic complaints in PTSD patients to help prevent exacerbation of physical symptoms leading to psychiatric decompensation and hospitalization.
